# Epidemiological Characteristics of 1196 Patients with Spinal Metastases: A Retrospective Study

**DOI:** 10.1111/os.12552

**Published:** 2019-11-22

**Authors:** Feng Wang, Hao Zhang, Li Yang, Xiong‐gang Yang, Hao‐ran Zhang, Ji‐kai Li, Rui‐qi Qiao, Yong‐cheng Hu

**Affiliations:** ^1^ Department of Bone Tumor Tianjin Hospital Tianjin China; ^2^ Graduate School Tianjin Medical University Tianjin China

**Keywords:** Epidemiological, Metastasis, Population, Spine

## Abstract

**Objectives:**

To describe the epidemiological characteristics of patients with spinal metastases between 2007 and 2019.

**Methods:**

Patients with spinal metastases were identified from several clinical centers in China between January 2007 and July 2019. Demographics, primary tumor types, spinal involvement, and Clinical indicators of each patient were reviewed.

**Results:**

A total of 1196 patients were included in this study, 717 males (59.95%) and 479 females (40.05%), with a male to female ratio of 1.50:1. Most patients (63.71%) were in the ages range of 50 to 69 years. The mean age was 58.6 ± 11.6 (range 13–89) years and the median age was 59.0 years. The average age of females was younger than that of males, and the difference was statistically significant. The proportion of male patients over 60 years old was higher than that of females, and the difference was statistically significant. The most common primary tumor was lung cancer (*n* = 437, 36.54%), followed by unknown origin (*n* = 194, 16.22%), kidney cancer (*n* = 78, 6.52%), breast cancer (*n* = 76, 6.35%), and liver/biliary cancer (*n* = 75, 6.27%). The most common primary tumor was lung cancer in both males and females, followed by unknown origin in males and breast cancer in females. There were 730 patients (61.04%) in the subgroup of the number<3; the highest level was lumbar vertebrae, with 250 patients (34.25%). The remaining 466 patients (38.96%) were included in the subgroup of the number ≥ 3; the highest level was tumor metastasis of multiple‐level of spine, with 334 patients (71.67%). Among the 1196 patients, spinal cord injury occurred in 54.01% of patients, 76.34% of patients developed moderate and above pain, 55.69% of patients had metastatic spinal cord compression, and only 26.67% of patients had a clear history of primary tumors.

**Conclusion:**

This study provided a relatively detailed description of epidemiological characteristics in spinal metastases in China, which could assist orthopaedic surgeons to understand the clinical characteristics of spinal metastases, and is of great significance in guiding clinical diagnoses and scientific research.

## Introduction

Improvements in clinical anti‐tumor multimodality therapies and palliative therapy have prolonged patients' survival, but the incidence of distant metastasis is still increasing^1^. The vertebral column is the third most frequent metastasis site after the lungs and liver, and accounts for approximately 50% of bone metastases^2^, ^3^, ^4^. Overall, 40% to 70% of patients with advanced neoplasia will eventually develop spinal metastases, and metastatic spinal cord compression (MSCC) will develop in 10% to 20% of these patients^5^, ^6^, ^7^. Of note, its main clinical manifestations are not only associated with severe pain but also with paralysis, sensory loss, sexual dysfunction, and urinary and fecal incontinency, especially when the neurologic elements are compressed, which are important factors leading to seriously reduced quality of life and even death^8^. The incidence rates and distributions of spinal metastases not only show significant geographic differences but also vary in age, gender, and ethnicity. Hence, understanding the epidemiologic characteristics of spinal metastases is fundamental and necessary for decisions regarding medical intervention and prediction of patients' prognosis.

In recent years, the number of epidemiological studies on spinal metastases has been gradually increasing worldwide. A spinal metastases epidemiology study that compared surgery trends across two decades and three continents concluded that surgical habits had been fairly consistent among countries worldwide and over time^9^. Moreover, the study of Sohn *et al*.^10^ was the first nationwide analysis of spine tumors, including metastatic spine tumors, in Asia. It reported epidemiology and healthcare utilization of 1600 patients with primary and metastatic spine tumors between 1 January 2009 and 31 December 2012. Horn *et al*.^11^ analyzed 333 patients <20 years old with spinal metastases undergoing spinal surgery from the Kid Inpatient Database in the United States and concluded that surgical treatment for spinal metastasis in the past decade has increased, although the complication rates, in‐hospital mortality, and length of stay remained stable.

However, some problems remain with epidemiological studies on spinal metastases in China. There are relatively few studies with large‐scale epidemiological analyses of spinal metastases. In addition, many studies focus on spinal tumors but not on spinal metastases specifically^12^, ^13^.

Hence, the purpose of this study is to: (i) describe the epidemiological characteristics of spinal metastases in China; (ii) focus attention on the distribution of age, gender, and primary tumor type in patients with spinal metastases; and (iii) provide guidance for clinical work and further research on spinal metastases.

## Patients and Methods

### *Patients Inclusion*

Patients with spinal metastases were identified from several clinical centers in China between January 2007 and July 2019. We retrieved and screened eligible cases through the hospital case management system by using disease codes, which were standardized according to the International Classification of Disease, Tenth Revision (ICD‐10), and patient information was obtained through analysis of inpatient medical records. A unified database was developed using an epidemiological method, including the patient's name, gender, age, primary tumor types, and level and number of involved vertebrae.

Inclusion criteria: (i) patients diagnosed with spinal metastases based on clinical symptoms, radiographic examinations, and/or histopathology; (ii) hematological malignancies including myeloma and lymphoma were also included; and (iii) patients whose observation indicators could be retrospectively analyzed.

Exclusion criteria: (i) patients with impaired spinal cord function due to severe spinal degenerative diseases; (ii) patients with other spinal diseases such as primary spinal tumors or spinal tuberculosis at the same time; and (iii) outpatients.

### *Recorded Indicators*

The following indicators were retrospectively recorded: (i) patient demographics, including age and gender; (ii) primary tumor types (origin of spinal metastases); and (iii) spinal involvement, including level and number of involved vertebrae (the sacral vertebrae and caudal vertebra were defined as one vertebra); and (iv) clinical indicators, including Frankel grade, visual analog scale (VAS) for pain, MSCC, and tumor history.

The Frankel grade classification provides an assessment of spinal cord function. There are five grades (A, B, C, D, and E) based on the degree of spinal cord injury^14^, as follows:

Grade A. Complete neurological injury: No motor or sensory function detected below the level of the lesion.

Grade B. Preserved sensation only: No motor function detected below the level of the lesion; some sensory function below the level of the lesion preserved.

Grade C. Preserved motor, nonfunctional: Some voluntary motor function preserved below the level of the lesion but too weak to serve any useful purpose; sensation may or may not be preserved.

Grade D. Preserved motor, functional: Functionally useful voluntary motor function below level of injury is preserved.

Grade E. Normal motor function: Normal motor and sensory function below level of lesion; abnormal reflexes may persist.

The VAS is a measure of pain intensity. It is a continuous scale comprised of a horizontal (called a horizontal visual analog scale) or vertical (called vertical visual analog scale), usually 10 cm or 100 mm length. For pain intensity, the scores can be from 0–10, which is determined by measuring the distance (mm) on the 10‐cm line between the “no pain” anchor and the patient's mark^15^.

Metastatic spinal cord compression is defined radiographically as an epidural metastatic lesion causing true displacement of the spinal cord from its normal position in the spinal canal^16^. It is an important source of morbidity (including paralysis and bowel and bladder disorders) in patients with systemic cancer.

Tumor history is defined as that the primary tumor has been clearly diagnosed before the diagnosis of spinal metastases.

### *Statistical Analysis*

The age and VAS were described using mean and median values, while gender, primary tumor, level and number of involved vertebrae, Frankel grade, VAS, MSCC, and tumor history were described using the composition ratios. The mean age of male and female patients was compared using Student *t*‐test. The difference in gender distribution among different age groups was statistically compared by χ^2^‐test. All statistical analyses were performed using IBM SPSS Statistics 22.0 (IBM, Armonk, NY, USA); a two‐tailed *P* < 0.05 was statistically significant.

## Results

### *Patient Demographics*

**Figure 1:**
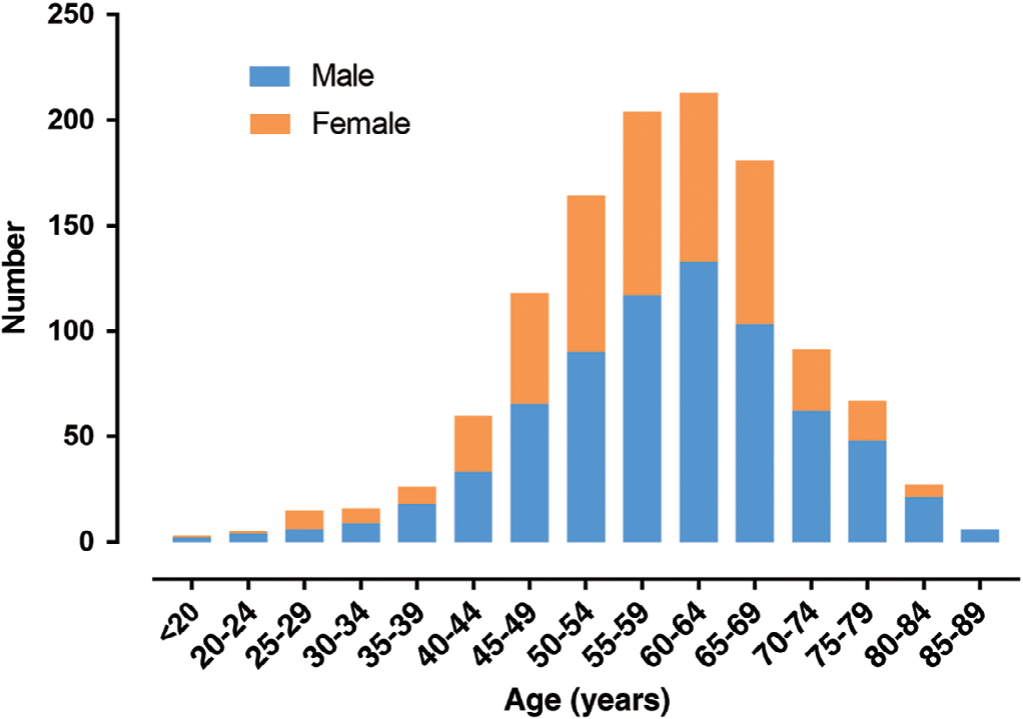
Distribution of gender and age in 1196 patients with spinal metastases. There were a total of 1196 patients in this study, including 717 males and 479 females, with a male to female ratio of 1.50:1; most patients were in the age range of 50–69 years.

A total of 1196 patients were included in this study, 717 males (59.95%) and 479 females (40.05%), with a male to female ratio of 1.50:1. Most patients (63.71%) were aged from 50 to 69 years. The mean age was 58.6 ± 11.6 (range 13–89) years and the median age was 59.0 years. The mean age of males and females was 59.4 ± 11.9 (range 16–89) years and 57.4 ± 11.1 (range 13–83) years, respectively, which showed that the onset time of spinal metastases was 2 years earlier in females than in males, and the difference was statistically significant (*t* = 2.96, *P* = 0.03). The proportion of male patients over 60 years old (52.02%) was 7.76% higher than that of females (44.26%), and the difference was statistically significant (χ^2^ = 6.926, *P* = 0.008). The distribution of age and gender in 1196 patients is shown in Table 1 and Fig. 1.

**Table 1:** Distribution of gender and age in 1196 patients with spinal metastases

Age	Male		Female		Total	
	N	%	N	%	N	%
<20	2	0.28	1	0.21	3	0.25
20‐24	4	0.56	1	0.21	5	0.42
25‐29	6	0.84	9	1.88	15	1.25
30‐34	9	1.26	7	1.46	16	1.34
35‐39	18	2.51	8	1.67	26	2.17
40‐44	33	4.60	27	5.64	60	5.02
45‐49	65	9.07	53	11.06	118	9.87
50‐54	90	12.55	74	15.45	164	13.71
55‐59	117	16.32	87	18.16	204	17.06
60‐64	133	18.55	80	16.70	213	17.81
65‐69	103	14.37	78	16.28	181	15.13
70‐74	62	8.65	29	6.05	91	7.61
75‐79	48	6.69	19	3.97	67	5.60
80‐84	21	2.93	6	1.25	27	2.26
85‐89	6	0.84	0	0.00	6	0.50
Total	717	100.00	479	100.00	1196	100.00

*N* = number of cases.

### *Primary Tumor Types*

**Figure 2:**
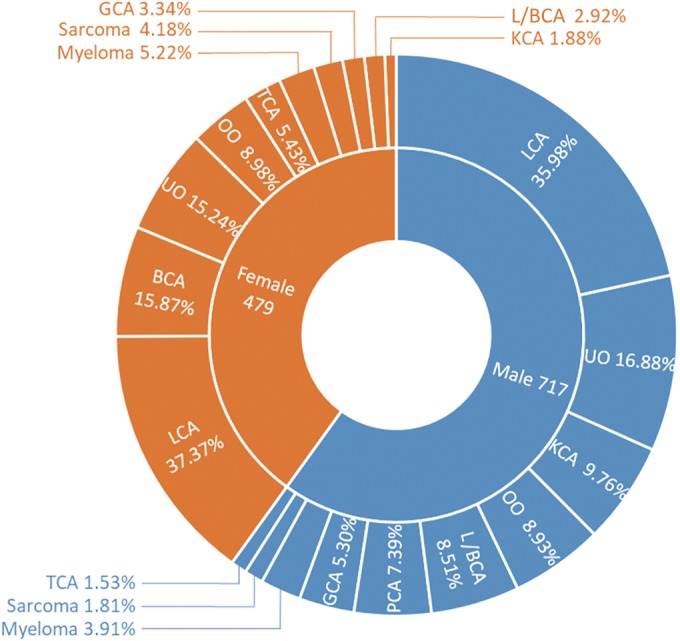
Distribution of gender and primary tumors in 1196 patients with spinal metastases. The most common primary tumor was lung cancer in both males and females, followed by unknown origin in males and breast cancer in females. BCA, breast cancer; GCA, gastrointestinal cancer; L/BCA, liver/biliary cancer; KCA, kidney cancer; LCA, lung cancer; OO, other origin; PCA, prostate cancer; TCA, thyroid cancer; UO, unknown origin.

The most common primary tumor was lung cancer (*n* = 437, 36.54%), followed by unknown origin (*n* = 194, 16.22%), kidney cancer (*n* = 78, 6.52%), breast cancer (*n* = 76, 6.35%), liver/biliary cancer (*n* = 75, 6.27%), gastrointestinal cancer (*n* = 53,4.43%), myeloma (*n* = 53, 4.43%), prostate cancer (*n* = 53, 4.43%), thyroid cancer (*n* = 37, 3.09%), sarcoma (*n* = 33, 2.76%), other origin (*n* = 107, 8.95%) such as esophageal cancer, lymphoma, and cervical cancer. The distribution of gender and primary tumors in 1196 patients is shown in Table 2 and Fig. 2.

**Table 2:** Distribution of gender and primary tumor in 1196 patients with spinal metastases

Primary tumor	Male		Female		Total	
	*N*	%	*N*	%	*N*	%
Lung cancer	258	35.98	179	37.37	437	36.54
Unknown origin	121	16.88	73	15.24	194	16.22
Kidney cancer	70	9.76	8	1.67	78	6.52
Breast cancer	0	0.00	76	15.87	76	6.35
Liver/biliary cancer	61	8.51	14	2.92	75	6.27
Gastrointestinal cancer	38	5.30	15	3.13	53	4.43
Myeloma	28	3.91	25	5.22	53	4.43
Prostate cancer	53	7.39	0	0.00	53	4.43
Thyroid cancer	11	1.53	26	5.43	37	3.09
Sarcoma	13	1.81	20	4.18	33	2.76
Other origin	64	8.93	43	8.98	107	8.95
Total	717	100.00	479	100.00	1196	100.00

*N* = number of cases.

According to the Tomita score, primary tumors can be divided into rapid growth tumors, moderate growth tumors, and slow growth tumors^17^. Among metastatic spine tumors in this study, 813 patients (67.98%) had rapid growth tumors, 129 patients (10.78%) had moderate growth tumors, and 254 patients (21.24%) had slow growth tumors.

### *Spinal Involvement*

The most common level of involved vertebrae was multi‐level of spine (*n* = 432, 36.12%), which means that tumor had involved 2 or more levels., followed by thoracic vertebrae (*n* = 316, 26.42%), lumbar vertebrae (*n* = 281, 23.50%), sacral vertebrae (*n* = 86, 7.19%), and cervical vertebrae (*n* = 81, 6.77%).

There were 730 patients (61.04%) in the subgroup of the number<3; the highest level was lumbar vertebrae, with 250 patients (34.25%). The remaining 466 patients (38.96%) were included in the subgroup of the number ≥ 3; the highest level was multiple‐level of spine, with 334 patients (71.67%). The distribution of involved vertebrae in 1196 patients with spinal metastasis is shown in Table 3.

**Table 3:** Distribution of involved vertebrae in 1196 patients with spinal metastasis

Involved vertebrae	<3		≥3		Total	
	N	%	N	%	N	%
Cervical vertebrae	67	9.18	14	3.01	81	6.77
Thoracic vertebrae	229	31.37	87	18.67	316	26.42
Lumbar vertebrae	250	34.25	31	6.65	281	23.50
Sacral vertebrae	86	11.78	0	0.00	86	7.19
Multiple‐ level of spine	98	13.42	334	71.67	432	36.12
Total	730	100.00	466	100.00	1196	100.00

*N* = number of cases.

### *Clinical Indicators*

There were 79 patients (6.60%) with Frankel grade A–B, 567 patients (47.41%) with Frankel grade C–D, and 550 patients (45.99%) with Frankel grade E. In total, there were 880 patients (54.01%) with Frankel grade A–D, which indicated that they a spine cord injury.

There were 283 patients (23.66%) with a VAS score of 0–3, 531 patients (44.40%) with a score of 4–6, and 382 patients (31.94%) with a score of 7–10 score. The mean VAS score was 5.2 ± 2.0 and the median score was 6.0 There were 913 patients (76.34%) aggregately with a VAS score of 4 or above, which indicated that they had developed moderate and above pain.

Among the 1196 patients, 666 patients (55.69%) had MSCC, and only 319 patients (26.67%) had a clear history of primary tumors.

## Discussion

This study included 717 males and 479 females with a male to female ratio of 1.50:1, and most patients were between the ages of 50 and 69 years. The average age of female patients was lower than that of male patients, and the difference was statistically significant. The proportion of male patients over 60 years old was higher than that of females, and the difference was statistically significant. For primary tumors, the most common site was lung cancer. When the number of involved vertebrae was fewer than three, the most level of which was lumbar vertebra. When the number of involved vertebrae was greater than three, the most level of which was multi‐level of spine. Among the 1196 patients, spinal cord injury occurred in 54.01% of patients, 76.34% of patients developed moderate and above pain, 55.69% of patients had MSCC, and only 26.67% of patients had a clear history of primary tumor.

### *Patient Demographics*

The study of Bollen *et al*.^18^ reported 1143 patients with spinal metastases, including 542 males (52%) and 501 females (48%), with an average age of 64.8 ± 12.5 years. Another study^19^ of spine metastases included 544 patients, including 287 males (52.8%) and 200 females (47.2%), with an average age of 63 years. Karhade *et al*.^20^ reported 732 patients with spinal metastases, including 426 males (58.2%) and 306 females (41.8%), with an average age of 61 years. In general, there were more men than women with spinal metastases, with an average age of approximately 60 years, which resembled the results of our study.

### *Primary Tumor Types*

Spinal metastases were mostly derived from epithelial tissue or glands, and a few were derived from mesenchymal tissue. Bollon *et al*.^18^ analyzed patients with spinal metastases from the Netherlands and found that the most common primary tumor was breast cancer, followed by lung cancer, prostate cancer, kidney cancer, and others. Similarly, an epidemiological study^9^ of spinal metastases across two decades and three continents showed that the most common primary tumor was breast cancer, followed by prostate cancer, lung cancer, kidney cancer, and others. A nationwide epidemiological study^21^ of adult Koreans with spinal metastases showed that the most common primary tumor was lung cancer, followed by liver cancer, breast cancer, colon cancer, and others. In general, the most common types of primary tumors varied greatly from country to country.

In this study, the most common primary tumor was lung cancer, followed by unknown origin, kidney cancer, breast cancer, liver/biliary cancer, and other. In males, it was followed by lung cancer, unknown origin, kidney cancer, liver/biliary cancer, prostate cancer, and others. In females, it was followed by lung cancer, breast cancer, unknown origin, thyroid cancer, and others. The results were similar to those for studies conducted in China and quite different from those reported in other countries. On the one hand, this may be due to the differences in the environment and ethnic origins, which resulted in different incidences of primary tumors. In China, lung cancer is the most common malignant tumor in both males and females. However, in Western countries, prostate cancer is the most common malignant tumor in males and breast cancer in females^22^. On the other hand, unknown origin accounted for 16.22% among the total patients in this study, which was quite different from 2%–4% in foreign countries^23^, ^24^, ^25^. This was related to the low economic level in some areas of China and the lack of knowledge about malignant tumors among the people. When some patients were diagnosed with spinal metastases, they would refuse further examinations to confirm primary tumors, leading to difficulty in identifying primary tumors.

### *Spinal Involvement*

The level and number of involved vertebrae in this study were similar to those in other studies. In the study of Bollen *et al*.^18^, the level of involved vertebrae was more common in multi‐level of spine, thoracic vertebrae and lumbar vertebrae, and the number of involved vertebrae was fewer than three in 517 patients (49.57%). Soon Bum *et al*.^26^ reported on 217 patients with spinal metastases, including 100 patients with thoracic metastasis, 65 patients with lumbosacral metastasis, and 32 patients with cervical metastasis.

### *Clinical Indicators*

The results for clinical indicators were similar to those of other studies^27^, ^28^. In this study, 73.33% of patients had no clear history of primary tumors when diagnosed with spinal metastases, and most of them saw a doctor because of local pain or MSCC. Therefore, for patients over 50 years old and without a history of tumors, there were no obvious cause of severe spinal pain, limb weakness, or sensory loss. When the conservative treatment effect was not obvious, it was necessary to alert the patients about the possibility of spinal metastases.

### *Limitations*

Several limitations of this study should be noted. First, approximately 50% of cases of spinal metastases would occur with symptoms, and approximately 10% of them would need surgical intervention^29^. Most of these patients without spine‐related symptoms were treated in oncology departments and rarely underwent surgical intervention. However, most of the patients included in this study incurred spine‐related symptoms and there were few patients who did not require surgical intervention. Therefore, the actual number of patients with spinal metastases in the population should be much higher than the number of patients included in this study. Second, the onset time, spine‐related symptoms, and the level and number of involved vertebrae in patients with spinal metastases were closely related to the biological behavior of primary tumors. This study only provides a general analysis and description of the patients and the spinal metastases characteristics. If the researchers focus on a single type of primary tumor, the results may be biased.

### *Conclusion*

The present study provides a relatively detailed description of epidemiological characteristics in spinal metastases in China, which could assist orthopaedic surgeons in understanding the clinical characteristics of spinal metastases and is of great significance in guiding clinical diagnoses and the scientific research.
